# Medium Optimization by Response Surface Methodology for Improved Cholesterol Oxidase Production by a Newly Isolated *Streptomyces rochei* NAM-19 Strain

**DOI:** 10.1155/2020/1870807

**Published:** 2020-03-31

**Authors:** Elsayed Ahmed Elsayed, Nayera Ahmed Abdelwahed

**Affiliations:** ^1^Bioproducts Research Chair, Zoology Department, College of Science, King Saud University, Riyadh 11451, Saudi Arabia; ^2^Department of Chemistry of Natural and Microbial Products, National Research Centre, Dokki, Cairo 12622, Egypt

## Abstract

Cholesterol oxidase is an alcohol oxidoreductase flavoprotein with wide biotechnological applications. The current work describes the isolation of a potential cholesterol oxidase producing streptomycete from Egyptian soil. The isolated strain produced cholesterol oxidase in submerged culture using a medium containing glucose, yeast extract, malt extract, and CaCO_3_ with the addition of cholesterol as an inducer. The isolated strain was identified as *Streptomyces rochei* NAM-19 based on 16S rRNA sequencing and phylogeny. Optimization of cholesterol oxidase production has been carried out using response surface methodology. The Plackett-Burman design method was used to evaluate the significant components of the production medium followed by Box-Behnken experimental design to locate the true optimal concentrations, which are significantly affecting enzyme production. Results showed that the predicted enzyme response could be closely correlated with the experimentally obtained production. Furthermore, the applied optimization strategy increased volumetric enzyme production by 2.55 times (65.1 U/mL) the initial production obtained before medium optimization (25.5 U/mL).

## 1. Introduction

Cholesterol oxidase, CO (EC. 1.1.3.6) enzyme is a monomeric oxidoreductase flavoenzyme, which catalyzes the oxidation of cholesterol to cholesterone and hydrogen peroxide. Cholesterol oxidase is an industrially important enzyme, which is used, in combination with related enzymes, in the clinical determination of serum cholesterol levels [1]. In addition, the CO enzyme is used in the biological processes involving conversion of different steroidal and nonsteroidal compounds [2]. Moreover, CO has been used as an insecticide in transgenic crop-pest management [3]. Meanwhile, many attempts have been made to reduce the cholesterol content of foods by the CO enzyme [4]. CO enzyme biosensors have found applications in the detection of cholesterol level in various samples [5] and have also scientific importance in investigating cell membrane interactions with cholesterol [6]. Due to its vast range of applications, the industrial importance and demand of CO have gained increased interest. CO enzyme has been produced by different microorganisms in submerged cultures, e.g., *Arthrobacter* sp., *Pseudomonas* sp., *Rhodococcus* sp., *Mycobacterium* sp., *Streptomyces* sp., *Nocardia*, and *Streptoverticillium* [7, 8]. Furthermore, some pathogenic bacteria were found to produce CO as a part of their mechanism to interfere with cell membrane integrity, hence enabling them to invade the host cells [9]. Accordingly, since CO enzyme has many applications in food and medical sectors, therefore, it is of great importance that the producing organisms should have the GRAS (generally regarded as safe) status [10]. Accordingly, search for new and safe microorganisms capable of producing such an industrially important enzyme has continued. *Streptomyces* spp. have been known as the best producers of CO with the least possible virulence or pathogenicity [11].

The commercial production of microbial valued products is generally performed in submerged cultivation systems [12–14]. The optimization of the production process depends mainly on two strategies to improve the volumetric production capabilities of the producing strain; (i) optimization of medium composition and (ii) improvement of the production process parameters [15]. The optimization of the composition of the production medium is generally achieved by optimization of the medium composition either by OFAT (one-factor-at-a-time) or by statistical optimization approaches [16]. Although OFAT optimization is traditional and simple to use, it is time and cost consuming and requires many experiments to be finalized [17]. Moreover, OFAT optimization does not consider the interaction between different factors investigated [18]. On the other hand, statistical medium optimization approaches have the advantages of enabling nutrient rebalancing and enrichment of essential medium components, while reducing unnecessary ones; i.e., they allow investigating the interaction between different factors [19]. Additionally, statistical approaches reduce the number of experiments to be performed, which is greatly reflected in the costs and thus the economy of the production process [16]. Generally, the statistical optimization of variable medium components produces a definite optimal response, either cell growth or volumetric production, which can be obtained through factorial design and the use of response surface methodology (RSM). RSM is a powerful mathematical tool for testing multiple process variables, which reduces experimental trials required to evaluate the significant components affecting the microbial process [20, 21].

The aim of the current investigation was to isolate and molecularly identify a potential actinomycete strain capable of producing CO. Furthermore, the statistical optimization design was adopted to optimize the composition of the production medium through application of the RSM approach. This was achieved by implementing both Plackett-Burman (PBD) and Box-Behnken (BBD) design methodologies. Finally, both unoptimized and statistically optimized medium compositions were compared in terms of total CO volumetric production.

## 2. Materials and Methods

### 2.1. Sample Collection and Isolation of Cholesterol Oxidase Producing Strains

Firstly, CO enzyme-producing microbial strains were isolated from different soil samples collected from El-Giza Governorate, Giza, Egypt. The isolation medium is composed mainly of mineral agar screening medium supplemented with cholesterol as the sole carbon source for growth. Serial dilutions were prepared from soil samples, from which loops were inoculated on the initial screening agar plates. CO-producing microbial colonies growing on the plate's surface appeared white and chalky with a characteristic soil smell, indicating cholesterol oxidization by the growing isolates. The potential of CO production was evaluated as follows: a loop full slant culture was suspended in sterile 0.85% NaCl solution. A portion of the suspension was spread on cholesterol enrichment medium containing the following (g/L): yeast extract, 10; KH_2_PO_4_, 0.05; NaNO_3_, 1; MgSO_4_, 0.5; cholesterol (dissolved in 1% Triton X-100), 2; and agar, 15. Plates were incubated at 32°C for 7 days. Colonies forming halos were streaked on CO indicator agar plates and incubated at 32°C for 2–4 days [11]. The development of intense brown pigmentation around the growing colonies indicates the production of CO. These colonies were selected and maintained on slants containing starch nitrate agar medium composed of the following (g/L) : starch 20; KNO_3_, 2; K_2_HPO_4_, 1; MgSO_4_.7H_2_O, 0.5; and agar, 20. After sporulation, spores were suspended in 20% (v/v) glycerol and were collected and stored at −20°C for subsequent experiments.

### 2.2. Identification of CO-Producing Isolate

The most potent actinomycete isolates were grown for 7 days on starch agar slants at 32°C. 2 ml of a spore suspension was inoculated into starch nitrate broth and incubated for 3 days on an incubator shaker (Innova 4080, New Brunswick Scientific, NJ, USA) at 200 rpm and 32°C to obtain heavy growth of vegetative cells (presporulation). The preparation of total genomic DNA was conducted in accordance with the methods described by Sambrook et al. [22]. PCR amplification of the 16S rRNA gene of the local actinomycete strain was conducted according to Edwards et al. [23]. Primers used in PCR amplification were the F27 primer (5-AGAGTTTGATCMTGGCTCAG-3) and the R1492 primer (5-TACGGGYTACCTTGTTACGACTT-3). Purification and sequencing of PCR products of the isolate were performed and DNA sequence similarity was compared with sequences deposited in the Gene Bank database using the BLAST program (http://www.ncbi.nlm.nih.gov). Multiple sequence alignment and molecular phylogeny were evaluated using BLAST software [24]. Spore morphology and spore chain were investigated by Scanning Electron Microscopy (SEM) (JEOL, JSM-5910, Japan). A plug of agar containing the culture was removed and fixed in glutaraldehyde vapor (2% v/v) at room temperature for 3 hours. Then, samples were dehydrated with increasing ethanol concentrations (50, 60, 70, 80, and 95%) for 15 min each and then twice with 100% ethanol (30 min/round). Ethanol was substituted with acetone and subjected to a critical point dryer (CPD7510, Polaron, Rang). The samples were sputter-coated with gold in an SPI-Module TM Sputter Coater (SPI Supplies, Division of Structure Probe Inc., USA) and observed by SEM.

### 2.3. Inoculum Preparation

Erlenmeyer flasks (250 mL) containing 100 mL of broth liquid medium were used to prepare the inoculum. The medium composed of the following (g/L): glucose, 20; yeast extract, 10; KH_2_PO_4_, 0.05; NaNO_3_, 1; and MgSO_4_, 0.5. Before sterilization, the pH of the medium was adjusted to 7.0, and then flasks were sterilized by autoclaving at 121°C and 1 atm. After cooling, flasks were inoculated with a loop of the actinomycete isolate previously maintained on starch nitrate agar slants. Inoculated flasks were incubated on a rotary shaker at 32°C and 200 rpm for 48 h, and the growing vegetative cells were used as standard inoculum.

### 2.4. Production Medium

The initial production medium was prepared according to Niwas et al. [25] in Erlenmeyer flasks containing 50 mL of medium. The medium composed of the following (g/L): glucose, 4; yeast extract, 4; malt extract, 10; CaCO_3_, 2; and cholesterol, 2. Cholesterol was used throughout the work as an inducer for CO enzyme production. Upon medium preparation, cholesterol was dissolved in 1% Triton X-100. The pH was adjusted at 7.0, and the flasks were autoclaved as previously described. Flasks were inoculated with 2 mL of vegetative cells from the inoculum prepared earlier. Inoculated flasks were incubated on a rotary shaker incubator at 200 rpm and 32°C. After the specified incubation time for each set of experimental trials, the mycelial growth was collected by centrifugation at 5000 rpm for 10 min. The cell-free supernatant was used for the determination of the CO enzyme.

### 2.5. Identification of Most Significant Nutrients by Plackett-Burman Design

Plackett-Burman design (PBD) was used to identify significant nutrients affecting CO production [26]. The four main variables investigated, i.e., glucose, yeast extract, malt extract, and CaCO_3_, were represented by *X*1, *X*2, *X*3, and *X*4, respectively. These variables were selected based on previous literature [25]. The variables were evaluated in two levels, high (+1) and low (−1) levels. Different experimental runs (12 runs) were performed according to the model. PBD experimental design is based on the first-order polynomial equation:

(1) $$\begin{array}{c} Y=\beta_{0}+\sum \beta_{i}\;X_{i}, \end{array}$$

where *Y* is the activity of CO, *β*_0_ is the coefficient of the model, *β*_*i*_ is the linear coefficient, and *X*_*i*_ is the levels of each of the independent factors. From the regression analysis, variables that were significant at or above 95% level and at probability value of *p* < 0.05 were considered to have a great impact on CO production and were further statistically optimized by BBD.

### 2.6. Box-Behnken Design Experiments

Following PBD medium optimization, the most significant factors affecting CO production were evaluated using BBD. Experiments involving four variables resulted in a combination of 27 experiments, 4 continuous factors, and 3 replicates at the center point. The variables tested were *X*1 (glucose), *X*2 (yeast extract), *X*3 (malt extract), and *X*4 (CaCO_3_). Each independent variable was coded in 3 levels (−1, 0, and +1). The response function (*Y*) representing CO activity was partitioned into linear, quadratic, and interactive components, which were represented using the second-order polynomial function as

(2) $$\begin{array}{c} Y=\beta_{0}+\sum \beta_{i}\;x_{i}+ßiixi^{2}+\sum \beta_{ij}\;X_{i}\;X_{j},\;i=1,2,3,...\text{,}k2, \end{array}$$

where *β*_*0*_, *β*_*i*_, *β*_*ii*_, and *β*_*ij*_ are the coefficients of intercept, linear, quadratic, and interactive terms, respectively, while *X*_*i*_ and *X*_*j*_ are the coded values of the four independent variables under study. The accuracy of the fitted model was justified through the analysis of variance (ANOVA) and the coefficient of *R*^2^. The significance of all terms in the polynomial model was judged statistically by computing the *F*-value at a probability (*p* value) of 0.05. Minitab software 17.0 was used for the regression of the experimental data and to generate the 2D contour plots, which were generated by keeping two-variable constants at 0 levels and varying the other variables within the experimental range. The whole set of experiments was performed in triplicate and the mean response was used for analysis.

### 2.7. Enzyme Assay

The activity of the extracellular CO enzyme was determined according to the method described by Inouye et al. [27]. Briefly, 0.1 mL of culture supernatant was added to 0.4 mL of 125 mM Tris-HCl buffer (pH 7.5). The mixture was incubated in a water bath at 37°C. After 3 minutes, 25 *µ*L of 12 mM of cholesterol in isopropanol solution was added to the mixture, and incubation proceeded for a further 30 minutes. Afterwards, 2.5 mL of absolute ethanol was added to the reaction medium, and then the amount of formed 4-cholesten-3-one was determined spectrophotometrically by measuring the absorbance at 240 nm. Reaction blanks were prepared by cholesterol solution with isopropanol. One unit of cholesterol oxidase activity (U) was defined as the amount resulting in the formation of 1 *µ*mol of 4-cholesten-3-one in 30 minutes at 37°C. The concentration of 4-colesten-3-one was calculated from a standard curve previously prepared with serial dilutions (10–100 *µ*g) of 4-cholesten-3-one dissolved in isopropanol.

## 3. Results and Discussion

### 3.1. Isolation, Identification, and Molecular Characterization of Cholesterol Oxidase Producing Strain

**Figure 1:**
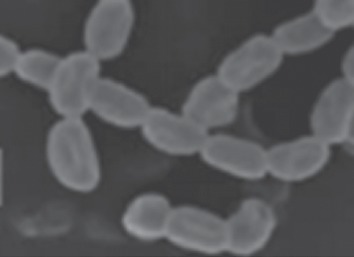
Spore chain morphology of *Streptomyces rochei* NAM-19 showing smooth surfaces in Rectiflexibiles (RF) chains by SEM (15 Kv × 10,000).

**Figure 2:**
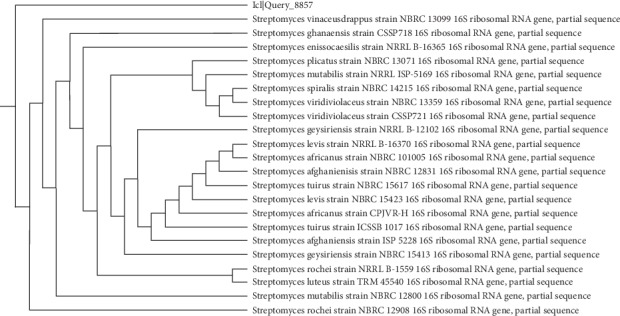
Neighbor-joining phylogenetic representation of the strains and their closest relatives based on 16S rRNA gene sequences of *S*. *rochei* NAM-19.

The first task of the work was to isolate an actinomycete strain capable of producing CO enzyme. Among different isolated colonies, the most active colony in terms of the highest CO activity was isolated and was further identified on a molecular basis. The isolated actinomycete formed extensive branching in both substrate mycelia and aerial hyphae, which differentiated into long straight chains (Rectiflexibiles type) carrying smooth-surfaced spores (Figure 1). The colony colour varies from white to grey on starch nitrate agar medium. BLAST software was used to compare the partial nucleotide sequence of the 16S rRNA gene of the CO-producing isolate with nucleotide databases found in NCBI webserver. Results showed higher similarity ranges (99.31–99.41%) with different *Streptomyces* strains, with an E-value of 0.0. From the phylogenetic tree (Figure 2), constructed based on molecular taxonomy and phylogeny, the isolated strain under study was closely related to many *Streptomyces* sp. [28]. Among nucleotides sequences of the 16S rRNA gene that were aligned, the similarity of the potent isolate was 99.4% to *Streptomyces rochei* NRRL 1559. Hence, the strain was identified as a novel *Streptomyces rochei* NAM-19, and the 16S rRNA sequence was deposited in the GenBank database under accession number MN630193.

### 3.2. Evaluation of the Most Significant Factors Affecting CO Activity

The optimization of key nutrients in fermentation medium for CO production by *S*. *rochei* NAM-19 using PBD was carried out in submerged cultures.  Table 1 presents different investigated variables with their high and low levels, as well as their obtained response in the activity of CO enzyme. It can be seen that the obtained CO activities ranged from 8.06 to 21.94 U/mL. Furthermore, the maximal enzyme production response was obtained in runs 1, 4, and 10 (21.46–21.94 U/mL). The variation reflected the significance of glucose, yeast extract, malt extract, and CaCO_3_ on CO production by the isolated strain. Maximal CO production was obtained in the 4^th^ run, which may be attributed to the presence of high levels of glucose, malt extract, and CaCO_3_ and low level of yeast extract. On the other hand, decreased CO production was correlated with inversed patterns of the four investigated medium components. It is generally known that combination of medium ingredients has a profound influence on the metabolic pathways of the producing organisms that regulate the production of different metabolites [29]. Various compounds such as cholesterol, yeast extract [30], potato starch, peptone, and malt extract [31] have been recorded to be substrates for an enhanced CO production.

The adequacy of the model was tested and parameters with statistically significant effects were identified using Fisher's (F) test for the analysis of variance (ANOVA). The analysis of variance for the selected factorial model showed that the model was significant with a Model *F*-value of 101.06 and *p* value of 0.000, which means that the above model is reliable enough to describe the quantitative relation between CO production and the four important medium components (Table 2).

The regression equation obtained from PBD was used to predict the factors affecting the CO activity response. The equation was expressed by *R*^2^ coefficient, which was about 98.30% successful in predicting the effects of the variables on CO production by *S*. *rochei* NAM-19. Therefore, all investigated factors were significant (*p* ≤ 0.05) and the model equation for enzyme production can be represented as

(3) $$\begin{array}{c} \text{CO activity}\left(\text{U/mL}\right)=9.70+1.808X1-1.096X2+0.271X3+0.391X4, \end{array}$$

where positive signs before the coefficient values, in case of glucose, malt extract, and CaCO_3_, indicate the significance of these factors at their high levels towards CO production. This indicated that CO production was enhanced by adding a higher concentration of these ingredients, whereas the negatively signed yeast extract has an inverse relationship with CO production (Table 3).

**Figure 3:**
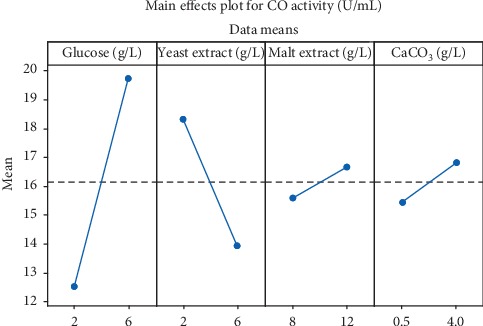
Effect of glucose, yeast extract, malt extract, and CaCO_3_ concentrations on the mean enzyme activity represented by the main effect plot.

Figure 3 represents the main effects of different factors on CO production obtained from PBD. The main effect enables the estimation of the influence of each factor on enzyme production. Both large positive or negative effects indicate that a variable has a large impact on production. From the main effect results, it can be noticed that glucose, malt extract, and CaCO_3_ have a positive influence on enzyme production, whereas yeast extract negatively affects CO production. Variables with positive impacts on CO production have been used at their high levels, while variables with a negative effect were kept at their low level for further optimization.

**Figure 4:**
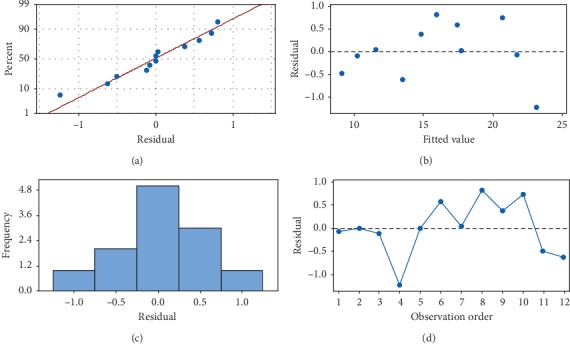
Residual plots for CO activity. (a) Normal Probability Plot. (b) Versus Fits. (c) Histogram. (d) Versus Order.

In order to confirm model adequacy, residual analysis plots (Figure 4) were generated. The normality of the data can be checked by plotting the normal probability plot (NPP) of the residuals. The normal probability plot is a graphical representation for assessing whether or not a data set is approximately normally distributed [32]. The residual is the difference between the observed and the predicted value (or the fitted value) from the regression. It could be seen that the experimental data points were approximately linear, suggesting normal distribution, which indicated that the model can be used to optimize the production of CO. Histograms of the residuals showed an almost symmetrical histogram (bell-shaped, i.e., the errors are normally distributed with mean zero). Regarding the plots of residuals versus the fitted values (predicted response), the residuals are scattered randomly about zero; i.e., errors have constant variance and all other points were found to fall in the range of +1 to −1.

**Figure 5:**
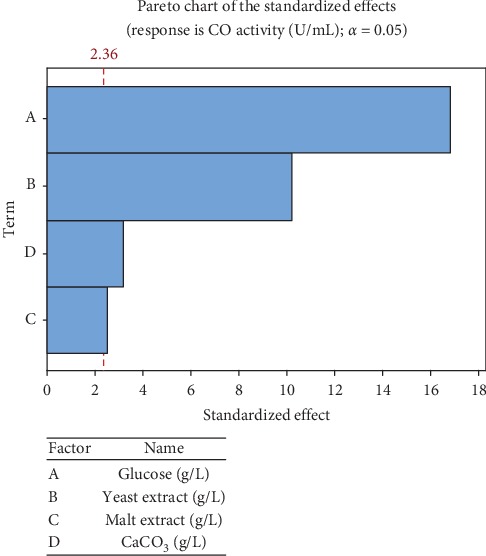
Pareto chart for the effect of medium nutrient components according to PBD.

**Figure 6:**
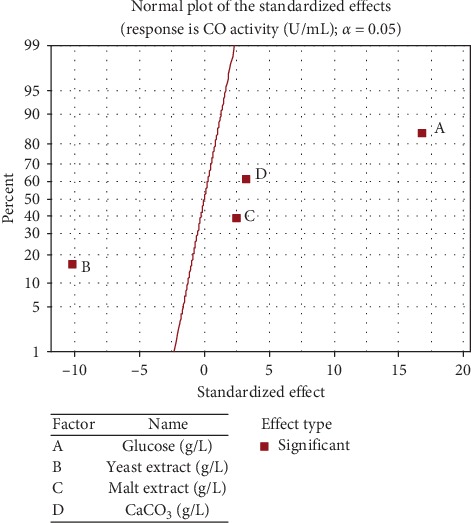
Normal plot of the standardized effects of components on CO enzyme activity.

Pareto chart (Figure 5) represents the estimated effects of variables on enzyme activity response in decreasing the order of magnitude. The length of each bar is proportional to the standardized effect. The vertical line can be used to judge which effects are statistically significant. Bars extending beyond this line correspond to statistically significant effects at a confidence level of 95% [33]. It can be seen that all independent variables had a significant effect towards enzyme activity response. Additionally, significant factors were further confirmed by the normal plotting of standardized effects (Figure 6). It can be seen that the three significant variables have higher percentages and are lying together on the right-hand side of the standardized effect line. Accordingly, the four variables were further considered for final optimization using BBD.

### 3.3. Response Surface Experimental Design Using BBD

The optimization of medium key components was further evaluated using RSM by BBD. This was performed to determine the true optimal concentrations of the key variables affecting CO enzyme production and to analyse the interactive effects of their concentrations. For these four variables, a design matrix of 27 runs with three levels (−1, low; 0, middle; +1, high) of each variable was constructed.  Table 4 presents the combinations of these 27 runs with their obtained enzyme activity responses. From these results, it can be seen that maximal enzyme activity response of 57.73 U/mL was obtained in run 18, which contained higher level of glucose (8 g/L), middle levels of yeast extracts and calcium carbonate (2 and 4 g/L, respectively), and lower level of malt extract (10 g/L).

The statistical significance of the model is determined by *F*-value obtained from ANOVA analysis (Table 5). A large Fisher's value (*F*-value = 7500.08) indicates that most of the variation can be explained by a regression equation, whereas a low *p* value (*p* < 0.005) indicates the statistical significance of the model. Regression coefficient (*R*^2^) came out to be 99.99%, the predicted *R*-squared (99.93%), and the adjusted *R*-squared (99.98%), which were in a reasonable argument with each other. Looking at the obtained *p* values for the model, results showed that the linear terms *X*1, *X*2, and *X*3, all quadratic terms *X*1^2^, *X*2^2^, *X*3^2^, and *X*4^2^, and cross terms *X*1 *∗* *X*2, *X*1 *∗* *X*3, *X*1 *∗* *X*4, *X*2 *∗* *X*4, and *X*3 *∗* *X*4 were highly significant.

Furthermore, Table 6 presents the regression analysis of BBD. It can be seen that the interaction between two variables can have either a synergetic effect, which is a positive coefficient increasing CO production, or an antagonistic effect, which is a negative coefficient indicating a decrease in CO production. Moreover, the analysis showed a significant negative quadratic effect of yeast extract and CaCO_3_, indicating that CO production increases with the increase of these parameters, then, reaches a maximum, and finally decreases at even higher concentrations of both variables (Table 6).

Generally, CaCO_3_ is used as a buffering agent in the production medium of most of the actinomycete strains, due to its high buffering capacity. However, the statistical optimization results showed that it is not significant. This could be attributed to the effect of Ca^+2^ ions on the morphology of the growing cells and consequently on CO production. It has been reported that addition of Ca^+2^ ions tends to decrease the probability of formation of pellets and enhances the formation of dispersed mycelia [34]. Moreover, the formed fewer pellets were of reduced size [35, 36]. This effect on the morphology of growing cells was reflected in the reduced production of peroxidase, protease, and oxidases by *Streptomyces* sp. However, we preferred not to remove CaCO_3_ from the production medium in order to benefit from its buffering capacity, which in turn provides the growing cells with their optimal required conditions.

Results obtained from BBD experimental runs were used to estimate the coefficients of the quadratic polynomial equation. The second-order polynomial equation characterizing relationships between CO production and different variables can be expressed as

(4) $$\begin{array}{c} \text{CO activity}\left(\text{U/mL}\right)=-16.44+5.212X1+1.695X2+5.742X3-0.039X4+0.4455X12-0.8187X22-0.1377X32-0.2956X42\\ +0.2100X1*X2-0.6544X1*X3+0.3750X1*X4+0.0187X2*X3+0.5828X2*X4-0.0750X4*X4. \end{array}$$

**Figure 7:**
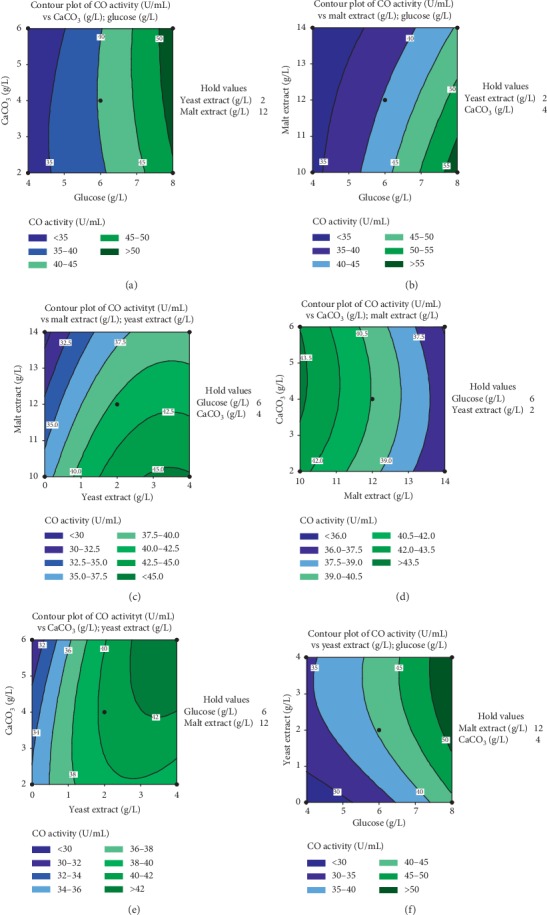
Contour plots for CO production as a function of (a) CaCO_3_ and glucose concentration, (b) glucose and malt extract concentration, (c) yeast extract and malt extract concentration, (d) malt extract and CaCO_3_ concentration, (e) yeast extract and CaCO_3_ concentration, and (f) yeast extract and glucose concentration.

Figures 7(a)–7(f) represent 2D contour plots of different relationships between independent and dependent variables of the model. It is clearly noticed that different contours indicate different interactions between the investigated variables. Generally, a circular contour plot means negligible interactions between the corresponding variables, while elliptical contours suggest the presence of a significant interaction between the corresponding variables. In our case, two variables were depicted in the contour plot, while the other two variables were fixed at zero levels (their preset middle concentration).  Figure 7(a) shows the effect of interaction between CaCO_3_ and glucose concentration on CO production. Increasing glucose concentration up to 8 g/L and CaCO_3_ from 3 to 6 (g/L) resulted in maximal CO production above 50 U/mL. However, below these values, there was a gradual decline in CO production. On the other hand, increasing CaCO_3_ concentration from 3.5 to 5 g/L and fixing malt extract concentration at 10 g/L increased CO production above 43.5 U/mL (Figure 7(d)). Furthermore, increasing malt concentration and decreasing CaCO_3_ concentration from these optimal levels resulted in a gradual decrease in CO production.

It may be also observed that higher yeast extract concentrations (3 to 4 g/L) combined with increasing CaCO_3_ concentration from 4 to 6 g/L resulted in an increase in enzyme production over 42 U/mL. On the other hand, below these optimal concentrations, a decrease in CO production was obtained (Figure 7(e)). Similar enhancement effects of higher levels of glucose and yeast extract showed a strong positive interaction effect on CO production as shown in Figure 7(f). From the contour plots in Figure 7(b) and 7(c) in Figure 6, it can be concluded that higher levels of glucose and yeast extract while keeping malt extract concentration at lower levels resulted in an improved CO production.

### 3.4. Interpretation of Process Optimization Curves

**Figure 8:**
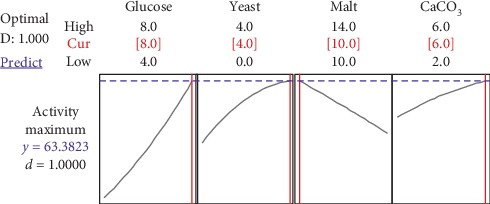
Process optimization curve for maximum enzyme activity.

RSM optimization is generally used to identify factor settings, which optimize the required response (CO production). In the present study, the goal for maximization of CO production by *S*. *rochei* NAM-19 was to obtain an enzyme production level, which is approximate to the statistically targeted concentration of 62.83 U/mL. Results showed that glucose, yeast extract, and CaCO_3_ concentrations lower than 8, 4, and 6 g/L, respectively, together with malt extract concentration above 10 g/L, did not satisfy this requirement. After RSM optimization experiments, the best combination of factor settings for achieving the desired response was found to be as follows (g/L): glucose, 8; yeast extract, 4; malt extract, 10; and CaCO_3_, 6. These concentrations produced the predicted response of 62.83 U/mL with a desirability score of 1 (Figure 8). In general, optimization plots are used to obtain the predicted response with higher desirability score, to lower-cost factor settings with near-optimal properties, and to study the sensitivity of response variables to changes in the factor settings [20, 21].

### 3.5. Validation of the Model

**Figure 9:**
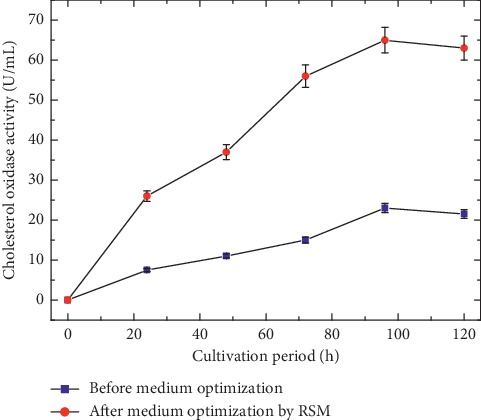
Comparative CO production in the cultivation medium before and after optimization through RSM by *S*. *rochei* NAM-19 strain.

Finally, to check the accuracy of the model, cultivation runs were performed to compare CO production under both initial medium composition and the final statistically optimized medium composition. Obtained results (Figure 9) showed that cultivation of *S. rochei* NAM-19 using the statistically optimized medium composition resulted in maximal production of 65.1 U/mL from the CO enzyme. This volumetric production level was closely related to the predicted response (62.8 U/mL). Furthermore, medium optimization increased maximal CO production by about 2.55 times the maximal CO production obtained using initial unoptimized medium (25.5 U/mL). Therefore, experimental runs prove that the model can be validated. RSM optimization has been applied to investigate the interrelationship between different medium components affecting the cell growth and production kinetics of many industrially microbial products [16, 20]. Furthermore, the obtained RSM optimization results are in good consistency with those previously reported for medium optimization by RSM for cholesterol oxidase production [37, 38]. The authors optimized medium composition by RSM approaches for CO production by *S*. *badius* and *S*. *lavendulae* NCIM 2499. The authors were able to achieve an increase in volumetric enzyme production by 2.48 and 2 times their initially used production media. Additionally, the newly isolated *Streptomyces rochei* NAM-19 showed initial CO production levels, which were comparable to those previously published in the literature by *Streptomyces* sp. [39].

## 4. Conclusion

During the current work, a new strain showing promising potential for cholesterol oxidase production was isolated from soil. The isolated strain was identified with the help of 16S rRNA and molecular taxonomy approaches as *S*. *rochei* NAM-19. The genomic sequence of the isolated strain was deposited in the NCBI database under the accession number MN630193. Furthermore, Plackett-Burman optimization showed that the initial concentration of glucose, yeast extract, malt extract, and CaCO_3_ significantly affected enzyme production. Additionally, Box-Behnken design revealed that the composition (g/L) of the key medium variables influencing enzyme production is as follows: glucose, 8; yeast extract, 4; malt extract, 10; and CaCO_3_, 6. The applied optimization model gave a predicted enzyme concentration of 62.83 U/mL with a desirability score of 1, which was very close to the experimentally obtained volumetric production level (65.1 U/mL). The application of RSM approach increased total enzyme production by about 2.55 times the initially unoptimized medium composition (25.5 U/mL).

## Figures and Tables

**Table 1:** PBD with enzyme activity response as affected by the four independent variables.

Run	*X*1 Glucose (g/L)	*X*2 Yeast extract (g/L)	*X*3 Malt extract (g/L)	*X*4 CaCO_3_ (g/L)	Response CO activity (U/mL)
1	6.0 (+1)	2.0 (−1)	12.0 (+1)	0.5 (−1)	21.732
2	6.0 (+1)	6.0 (+1)	8.00 (−1)	4.0 (+1)	17.713
3	2.0 (-1)	6.0 (+1)	12.0 (+1)	0.5 (−1)	10.081
4	6.0 (+1)	2.0 (−1)	12.0 (+1)	4.0 (+1)	21.940
5	6.0 (+1)	6.0 (+1)	8.00 (−1)	4.0 (+1)	17.713
6	6.0 (+1)	6.0 (+1)	12.0 (+1)	0.5 (−1)	18.000
7	2.0 (-1)	6.0 (+1)	12.0 (+1)	4.0 (+1)	11.597
8	2.0 (-1)	2.0 (−1)	12.0 (+1)	4.0 (+1)	16.761
9	2.0 (-1)	2.0 (−1)	8.00 (−1)	4.0 (+1)	15.245
10	6.0 (+1)	2.0 (−1)	8.00 (−1)	0.5 (−1)	21.460
11	2.0 (-1)	6.0 (+1)	8.00 (−1)	0.5 (−1)	8.6070
12	2.0 (-1)	2.0 (−1)	8.00 (−1)	0.5 (−1)	12.870

**Table 2:** Analysis of variance of CO activity versus glucose, yeast extract, malt extract, and CaCO_3_.

Source	DF	Adj. SS	Adj. MS	*F*-value	*p* value
Model	4	223.723	55.931	101.06	0.000
Linear	4	223.723	55.931	101.06	0.000
Glucose (*X*1)	1	156.942	156.942	283.56	0.000
Yeast extract (*X*2)	1	57.628	57.628	104.12	0.000
Malt extract (*X*3)	1	3.524	3.524	6.37	0.040
CaCO_3_ (*X*4)	1	5.629	5.629	10.17	0.015
Residual error	7	3.874	0.553		
Lack-of-fit	6	3.874	0.646		
Pure error	1	0.000	0.000		
Corrected total	11	227.597			
Model summary	S	R-seq.	R-seq. (Adjusted)	R-seq. (Predicted)	
	0.743952	98.30%	97.33%	95.00%	

**Table 3:** Regression analysis of PBD with model coefficients and significance of the regression coefficient for CO activity.

Term	Effect	Coefficient	SE coefficient	*T*-value	*p* value
Constant		9.700	1.280	7.570	0.000
*X*1	3.616	1.808	0.107	16.84	0.000
*X*2	−2.191	−1.096	0.107	−10.20	0.000
*X*3	0.542	0.271	0.107	2.500	0.040
*X*4	0.783	0.391	0.123	3.19	0.015

**Table 4:** BBD representing the predicted CO production by *S*. *rochei* NAM-19. Levels of coded variables are in parenthesis.

Run	*X*1 Glucose (g/L)	*X*2 Yeast extract (g/L)	*X*3 Malt extract (g/L)	*X*4 CaCO_3_ (g/L)	Response CO activity (U/mL)
1	4 (−1)	0 (−1)	12 (0)	4 (0)	26.300
2	8 (+1)	0 (−1)	12 (0)	4 (0)	43.600
3	4 (−1)	4 (+1)	12 (0)	4 (0)	34.040
4	8 (+1)	4 (+1)	12 (0)	4 (0)	54.000
5	6 (0)	2 (0)	10 (−1)	2 (−1)	42.400
6	6 (0)	2 (0)	14 (+1)	2 (−1)	36.000
7	6 (0)	2 (0)	10 (−1)	6 (+1)	43.440
8	6 (0)	2 (0)	14 (+1)	6 (+1)	35.840
9	4 (−1)	2 (0)	12 (0)	2 (−1)	33.640
10	8 (+1)	2 (0)	12 (0)	2 (−1)	49.240
11	4 (−1)	2 (0)	12 (0)	6 (+1)	31.100
12	8 (+1)	2 (0)	12 (0)	6 (+1)	52.700
13	6 (0)	0 (−1)	10 (−1)	4 (0)	36.300
14	6 (0)	4 (+1)	10 (−1)	4 (0)	45.200
15	6 (0)	0 (-1)	14 (+1)	4 (0)	29.140
16	6 (0)	4 (+1)	14 (+1)	4 (0)	38.340
17	4 (−1)	2 (0)	10 (−1)	4 (0)	33.880
18	8 (+1)	2 (0)	10 (−1)	4 (0)	57.730
19	4 (−1)	2 (0)	14 (+1)	4 (0)	32.120
20	8 (+1)	2 (0)	14 (+1)	4 (0)	45.500
21	6 (0)	0 (−1)	12 (0)	2 (−1)	34.200
22	6 (0)	4 (+1)	12 (0)	2 (−1)	38.600
23	6 (0)	0 (−1)	12 (0)	6 (+1)	29.976
24	6 (0)	4 (+1)	12 (0)	6 (+1)	43.700
25	6 (0)	2 (0)	12 (0)	4 (0)	41.500
26	6 (0)	2 (0)	12 (0)	4 (0)	40.300
27	6 (0)	2 (0)	12 (0)	4 (0)	39.500

**Table 5:** Analysis of variance of BBD for CO production model using *S*. *rochei* NAM-19.

Source	DF	Adj. SS	Adj. MS	*F*-value	*p* value
Model	14	1586.49	113.21	7500.08	0.000
Linear	4	8.72	2.181	144.34	0.000
*X*1	1	6.36	6.360	420.90	0.000
*X*2	1	0.88	0.878	58.19	0.000
*X*3	1	4.31	4.313	285.44	0.000
*X*4	1	0.00	0.000	0.03	0.870
Square	4	110.93	27.733	1835.51	0.000
*X*1^2^	1	16.93	16.933	1120.70	0.000
*X*2^2^	1	57.20	57.200	3785.77	0.000
*X*3^2^	1	1.62	1.617	107.04	0.000
*X*4^2^	1	7.46	7.457	493.51	0.000
2-Way interaction	6	61.34	10.224	676.67	0.000
*X*1 *∗* *X*2	1	2.82	2.822	186.80	0.000
*X*1 *∗* *X*3	1	27.41	27.405	1813.80	0.000
*X*1 *∗* *X*4	1	9.00	9.000	595.66	0.000
*X*2 *∗* *X*3	1	0.02	0.022	1.49	0.246
*X*2 *∗* *X*4	1	21.73	21.734	1438.47	0.000
*X*3 *∗* *X*4	1	0.36	0.360	23.83	0.000
Residual error	12	0.18	0.015		
Pure error	2	0.00	0.000		
Corrected total	26	1586.68			
Model summary	S	R-seq.	R-seq. (Adjusted)		R-seq. (predicted)
	0.122920	99.99%	99.98%		99.93%

**Table 6:** Regression analysis of BBD with model coefficients for total CO production by *S*. *rochei* NAM-19.

Term	Effect	Coefficient	SE coefficient	*T*-value	*p* value
Constant		−16.44	2.63	−6.26	0.000
*X*1	10.423	5.212	0.254	20.52	0.000
*X*2	3.390	1.695	0.222	7.62	0.000
*X*3	11.485	5.742	0.340	16.89	0.000
*X*4	−0.078	−0.039	0.235	−0.17	0.870
*X*1^2^	0.8909	0.4455	0.0133	33.48	0.000
*X*2^2^	−1.6375	−0.8187	0.0133	−61.53	0.000
*X*3^2^	−0.2753	−0.1377	0.0133	−10.35	0.000
*X*4^2^	−0.5912	−0.2956	0.0133	−22.22	0.000
*X*1*X*2	0.4200	0.2100	0.0514	13.67	0.000
*X*1*X*3	−1.3087	−0.6544	0.0514	−42.59	0.000
*X*1*X*4	0.7500	0.3750	0.0514	24.41	0.000
*X*2*X*3	0.0375	0.0187	0.0514	1.22	0.246
*X*2*X*4	1.1655	0.5828	0.0514	37.93	0.000
*X*3*X*4	−0.1500	−0.0750	0.0514	−4.88	0.000

## Data Availability

All data generated in this current work are included in the Results and Discussion sections.
